# Factors Associated With Differences in the Recommended Versus Actual Discharge Location After Stroke

**DOI:** 10.1016/j.apmr.2026.04.005

**Published:** 2026-04-09

**Authors:** Kimberly J. Waddell, Ruiqi Yan, Lin Xu, Stephen Hampton, Robert E. Burke, Rujula Upasani, Kiersten M. McCartney, Mucio Kit Delgado

**Affiliations:** a Department of Physical Medicine and Rehabilitation, Perelman School of Medicine, University of Pennsylvania, Philadelphia, PA; b Corporal Michael J. Crescenz VA Meical Center, Philadelphia, PA; c Penn Medicine Nudge Unit, Center for Health Care Trnsformation and Innovation, University of Pennsylvania Health System, Philadelphia, PA; d Leonard Davis Institute of Health Economics, University of Pennsylvania, Philadelphia, PA; e Dvision of General Internal Medicine, Perelman School of Medicine, University of Pennsylvania, Philadelphia, PA; f Division of Hospital Medicine, Perelman School of Medicine, University of Pennsylvania, Philadelphia, PA; g College of Medicine, Drexel University, Philadelphia, PA; h Department of Emergency Medicine, Perelman School of Medicine, University of Pennsylvania, Philadelphia, PA.

**Keywords:** Discharge, Postacute, Rehabilitation, Stroke

## Abstract

**Objectives:**

Discharge planning during an acute stroke hospitalization requires a coordinated effort to ensure patients receive the most appropriate posthospital care. We identified and described discrepancies in recommended versus actual postdischarge care after stroke.

**Design:**

This retrospective cohort study used the final acute hospital discharge recommendation by physical and occupational therapy as the recommended discharge disposition. We identified those who were discharged to a different care setting than was recommended and characterized sociodemographic, clinical, and facility factors associated with this discrepancy using a multinomial logistic regression.

**Setting:**

Acute care.

**Participants:**

Individuals hospitalized for an ischemic or hemorrhagic stroke between January 1, 2018, and December 31, 2024. The cohort (N=7545) comprised stroke hospitalizations.

**Interventions:**

Not applicable.

**Main Outcome Measures:**

The recommended and actual discharge disposition.

**Results:**

TThe mean (SD) age was 67 (14) years, 45.2% White and 42.0% Black. Overall, 1182 (15.6%) discharges were discrepant: 950 (80.4%) went to a lower intensity care setting, and 232 (19.6%) went to a higher intensity setting. The strongest associations with a discrepant discharge were a Sunday discharge (odds ratio [OR], 1.53; 95% CI, 1.10–2.11), Medicare fee-for-service (OR, 1.43; 95% CI, 1.14–1.79), or Medicare Advantage insurance (OR, 1.34; 95% CI, 1.08–1.68), and dependent prestroke ambulation (OR, 1.38; 95% CI, 1.14–1.68). These were also the factors most associated with increased odds of discharge to a lower intensity setting. A severe stroke (OR, 0.80; 95% CI, 0.67–0.96) and physiatry consult (OR, 0.75; 95% CI, 0.64–0.89) were associated with significantly lower odds of a discrepant discharge. Hemorrhagic stroke, increased function during admission, and greater social vulnerability were associated with increased odds of discharge to a higher intensity setting.

**Conclusions:**

The factors most associated with differences in recommended versus actual postacute care were unrelated to clinical need. Addressing these underlying factors will help ensure patients receive the recommended care to maximize outcomes. Archives of Physical Medicine and Rehabilitation 2026;107:2330–9

Stroke is a leading cause of complex, long-term disability.^1,2^ A stroke induces wide-ranging impairments across multiple body structures, such as cognitive, motor, and sensory systems.^3^ These complex impairments require multidisciplinary in-hospital care and coordinated discharge planning to ensure patients receive the most clinically appropriate postacute care to maximize outcomes and prevent hospital readmissions.^4^ A key knowledge gap is how many individuals do not receive the postdischarge care that is recommended by their acute hospital team. Most prior work focused on distinguishing those more likely to require postacute rehabilitation in a skilled nursing facility (SNF), compared with an inpatient rehabilitation facility (IRF).^5–7^ This is because care received in an IRF is associated with significantly greater functional improvement and lower hospital readmission rates, relative to SNF and home health (HH), but is more costly.^5,6,8,9^ These studies, however, do not capture whether the actual discharge aligned with what the care team recommended. This is crucial for evaluating the discharge plan’s execution and identifying opportunities to improve the planning process, ultimately ensuring patients receive appropriate care to maximize their long-term outcomes.

The purpose of this study was to examine the degree to which a patient’s discharge setting aligned with the recommended discharge setting poststroke, and the multilevel factors that are associated with a discrepant discharge. Furthermore, we examined the direction of discrepant discharges, defined as discharging to a more or less intensive setting than what was recommended by the care team. By characterizing the prevalence and elucidating the drivers and directionality of discrepant discharges, this study offers initial evidence for developing interventions that optimize discharge planning poststroke.

## Methods

This retrospective cohort study included adults who were admitted to a large academic health system with a primary discharge diagnosis of ischemic or hemorrhagic stroke between January 1, 2018, and December 31, 2024. The academic health system includes 6 acute hospitals from 2 states. This health system included IRF, SNF, and HH services within the health system, as well as multiple options for postacute care within the metropolitan area but outside of the health system. The Institutional Review Board at the University of Pennsylvania approved this study with a waiver of informed consent.

### Usual discharge planning process

Because two-thirds of individuals with stroke will receive rehabilitation services posthospitalization, physical (PT) and occupational therapists (OT) make discharge recommendations based on complex clinical reasoning, patient factors, and rehabilitation needs.^10^ This recommendation may change during an acute admission. A specialist consult, such as physiatry, may either confirm or modify the discharge recommendation, especially in cases where a patient may be appropriate for an IRF. For those recommended for a facility discharge, the bed availability at the next level of care and insurance authorization can delay or change the discharge placement.^11,12^ All of these steps occur in a matter of days, in a busy hospital environment, with limited resources and time. The final discharge recommendation balances clinical need, logistical reality, and patient preference. We sought to include relevant variables from each step of this process in our analysis.

## Cohort construction

This visit-level analysis included individuals with (1) a primary ICD-10 discharge diagnosis of an ischemic or hemorrhagic stroke (supplemental table S1, available online only at http://www.archives-pmr.org/); (2) discharged home, home with HH, an SNF, or an IRF; (3) were evaluated by PT and OT during the hospitalization; and (4) PT and OT recommended the same discharge location, indicating clinical alignment within the rehabilitation team. Patients were excluded if they (1) died during hospitalization; (2) transferred to a different acute hospital, hospice, or a long-term acute care facility; and (3) did not have a valid address in the electronic health record (EHR), limiting our ability to examine geographic variables in the analysis.

Data were extracted from the EHR using the Epic Clarity database. The PT and OT discharge recommendations were extracted for each admission using EHR flowsheet data. We extracted all discharge recommendations for every hospitalization to examine how PT and OT recommendations changed over the course of admission. In the adjusted analyses, the final documented PT and OT recommendation before hospital discharge was used as the recommended discharge disposition.

### Covariates

Sociodemographic variables included age, sex, race, ethnicity, insurance type, rurality, social vulnerability index (SVI), home layout, and preadmission mobility level. Insurance type was classified as commercial/private, Medicare fee-for-service, Medicare Advantage, or Medicaid. The commercial/private group was the reference group, enabling comparisons among Medicare and Medicaid beneficiaries with known variations in postacute care use and outcomes.^13,14^ Rurality was determined using the United States Department of Housing and Urban Development’s Urbanization Perceptions Small Area Index.^15^ Individuals were classified as urban, suburban, or rural residents. The SVI is a percentile-based measure of social vulnerability at the county level, ranging from 0 to 1, with higher scores indicating greater vulnerability.^16,17^

The clinical variables included type of stroke (ischemic or hemorrhagic), length of acute hospitalization, initial stroke severity (Stroke Administrative Severity Index), Activity Measure for Postacute Care (AM-PAC) 6-Clicks activities of daily living (ADL) and mobility scores, physiatry consultation during admission, and admission to the intensive care unit. The Stroke Administrative Severity Index is a validated measure that uses administrative data from the hospitalization to quantify initial stroke severity.^18,19^ The Stroke Administrative Severity Index ranges from 0 to 56, with higher scores indicating a more severe stroke. Consistent with prior work, the scores were categorized as either mild (0 points), moderate (1–6 points), or severe stroke (≥7 points).^18^ The AM-PAC 6-Clicks quantifies the amount of assistance needed to perform ADLs and mobility, with scores ranging from 6 to 24, with lower scores indicating more severe functional impairment. The AM-PAC 6-Clicks assessment scores predict hospital discharge poststroke and were used as a measure of function in this study.^20^

We also included temporal and facility variables in the analysis. This was motivated by potential differences in stroke discharge by day of the week or season, as well as facility-based factors that may pressure inpatient teams to accelerate discharges because of higher patient volume. There is mixed evidence for a “weekend effect”—that patients have worse outcomes during the weekend versus weekday—in stroke care.^21,22^ There is potential for a more fragmented discharge during the weekend, but this has not been previously investigated. As a result, we included discharge day of the week, calendar year, and season, categorized as winter (January-March), spring (April-June), summer (July-September), and fall (October-December) in the analysis. We included daily emergency department (ED) boarding hours as a measure of hospital pressure. This is calculated as the duration of time between when an order was placed for hospital admission in the ED and the patient’s ED departure. Boarding hours are one measure of ED overcrowding and are associated with worse patient care, clinical efficiency, worse patient outcomes, and higher health care costs.^23–25^ As ED boarding hours increase, there may be greater hospital pressure to increase bed availability, which may rush the discharge process.

### Outcomes

The primary outcome was a binary variable indicating if the actual discharge, extracted from the EHR, aligned with the recommended PT/OT discharge (yes/no). A secondary outcome was whether the discrepancy was for a more- or less intensive rehabilitation setting than what was recommended. An IRF was considered the most intensive discharge setting, followed by SNF, home with HH, and then home. For example, if a patient was recommended or discharged to an SNF, but their actual discharge was to an IRF, this was classified as a more intensive discharge. If a patient was recommended to discharge to SNF, but went home, this was classified as a less intensive discharge.

### Statistical analysis

In unadjusted analyses, we explored how discharge recommendations evolved over the duration of admission by plotting the initial PT and OT recommendation, discharge PT and OT recommendation, and the actual discharge disposition. For patients who were seen once by PT and OT, the single recommendation was used for both initial and discharge recommendations.

To examine factors associated with a discrepant discharge poststroke, we fit a generalized logistic regression model that included a patient and acute hospital random effect and the covariates listed above. We included calendar year in the model to account for trends in discharge disposition over time, as well as the onset of the COVID-19 pandemic in 2020. We applied a multistage approach to inform our adjustment for the COVID-19 pandemic. First, we visually examined the unadjusted trends in discharge disposition by year (supplemental fig S1, available online only at http://www.archives-pmr.org/). Next, we completed a Cochran-Armitage test for trend, which demonstrated that there was a significant change in the proportion of patients who were discharged to HH and IRF by year (*P*<.001), but not SNF or home. As a result, we included the calendar year to both account for the COVID-19 pandemic as well as sustained changes in discharge disposition after 2020. In the primary analysis, we used mean substitution for missing AM-PAC scores and SVI values. There were 111 (1.5%) missing AM-PAC ADL scores, 72 (1.0%) missing mobility scores, and 361 (4.8%) missing SVI values. We completed a sensitivity analysis that allowed for missing scores to examine the robustness of our findings. Model estimates were exponentiated to obtain odds ratios and 95% CIs.

To examine the secondary outcome of direction of discrepant discharges, we fit a multinomial logistic regression model with a patient and acute hospital random effect and the same covariates as the primary analysis. In this model, the 3 possible outcomes were (1) a matched discharge; (2) actual discharge to a more intensive setting than what was recommended; and (3) actual discharge to a less intensive setting than what was recommended. The matched discharge was the reference level. All analyses were completed in R, version 4.3.0.^a^

## Results

The final cohort comprised 7545 acute stroke hospitalizations and 6998 unique patients (fig 1). The cohort had a mean (SD) age of 67 (14) years, was 49.4% women, 45.2% White, and 42.0% Black (table 1). The mean (SD) hospital length of stay was 7.9 (10.9) days, and 3074 (40.7%) received a physiatry consultation during their admission. Overall, 1182 (15.6%) discharges were discrepant: 950 (80.4%) went to a lower intensity and 232 (19.6%) went to a higher intensity setting. The average time from admission to the first recorded AM-PAC ADL score was 53.9 hours, and 48.2 hours for the AM-PAC mobility score.

The unadjusted trends in discharge recommendations during acute admission are presented in figure 2. Overall, 3696 (48.9%) were recommended for discharge to an IRF, but only 3107 (41.2%) were discharged to an IRF. A total of 982 (13.1%) were recommended for discharge to an SNF, and 1096 (14.5%) were discharged to an SNF. Similarly, 1398 (18.5%) were recommended for HH, and 1812 (24.0%) were discharged with HH. Lastly, 1469 (19.5%) were recommended for discharge home, and 1530 (20.3%) were discharged home.

Results from the adjusted primary analysis demonstrated the strongest associations with a discrepant discharge were hospital discharge on a Sunday, relative to Monday (adjusted odds ratio [aOR], 1.53; 95% CI, 1.10–2.11; *P*=.01), Medicare fee-for-service (aOR, 1.34; 95% CI, 1.08–1.68; *P*=.009) and Medicare Advantage insurance (aOR, 1.43; 95% CI, 1.14–1.79; *P*=.002), relative to private/commercial, and nonindependent prestroke ambulation (aOR, 1.38; 95% CI, 1.14–1.68; *P*=.001), relative to independent prestroke ambulation (fig 3, supplemental table S2, available online only at http://www.archives-pmr.org/). An increasing length of hospitalization (in d) was also significantly associated with increased odds for a discrepant discharge (aOR, 1.01; 95% CI, 1.00–1.01; *P*=.03) and discharge in calendar year 2020, relative to 2018 (aOR, 1.47; 95% CI, 1.15–1.90; *P*=.002). In contrast, a more severe stroke (aOR, 0.80; 95% CI, 0.67–0.96; *P*=.02) and receiving a physiatry consultation were both associated with significantly lower odds of a discrepant discharge (aOR, 0.75; 95% CI, 0.64–0.89; *P*=.001). These results were consistent in sensitivity analyses (supplemental table S3, available online only at http://www.archives-pmr.org/).

In secondary analyses, several significant factors were associated with increased odds of discharge to a lower intensity setting, compared with those who had a matched discharge: Medicare fee-for-service insurance (aOR, 1.48; 95% CI, 1.15–1.90; *P*=.002), Medicare Advantage insurance (aOR, 1.56; 95% CI, 1.21–2.02; *P*<.001), nonindependent prestroke ambulation (aOR, 1.37; 95% CI, 1.11–1.69; *P*=.003), and Sunday discharge (aOR, 1.55; 95% CI, 1.08–2.23; *P*=.02), relative to Monday. Calendar year 2020 (aOR, 1.85; 95% CI, 1.38–2.48; *P*<.001), 2021 (aOR, 1.38; 95% CI, 1.01–1.87; *P*=.04), and 2022 (aOR, 1.56; 95% CI, 1.14–2.13; *P*=.005) were associated with significantly higher odds of discharge to a lower intensity setting, relative to calendar year 2018 (fig 4 and supplemental table S4, available online only at http://www.archives-pmr.org/).

In contrast, compared with those with a matched discharge, individuals had significantly higher odds of discharge to a higher intensity setting than what was recommended if they had a hemorrhagic, relative to ischemic, stroke (aOR, 1.58; 95% CI, 1.07–2.33; *P*=.02), had higher AM-PAC ADL (aOR, 1.07; 95% CI, 1.01–1.13; *P*=.03), and AM-PAC mobility scores (aOR, 1.16; 95% CI, 1.10–1.22; *P*<.001) indicating less functional impairment, and increased social vulnerability (aOR, 2.18; 95% CI, 1.17–4.08; *P*=.02). Individuals had significantly lower odds of discharge to a higher intensity setting if they received a physiatry consult during the acute hospitalization (aOR, 0.33; 95% CI, 0.21–0.52; *P*<.001). Higher hospital pressure on the day of discharge was also associated with significantly lower odds of discharge to a higher intensity setting (Q4 boarding h aOR, 0.52; 95% CI, 0.29–0.91; *P*=.02), compared with the lowest quartile (fig 5, supplemental table S4).

## Discussion

We found that 15.6% of individuals with stroke were not discharged to the care setting recommended by the acute therapy team, and >80% of these were discharged to a lower intensity care setting than recommended. The strongest associations with discrepant discharges included nonmodifiable variables, such as insurance type, day of discharge, and prestroke mobility. After a stroke, the acute hospital discharge is often the first of many care transitions and is uniquely vulnerable given the acuity of illness.^26,27^ Discharging to the setting that was recommended by the care team is important for the continuity of care and ensuring that patients receive the medical and rehabilitation care needed to maximize outcomes.^26^ To the best of our knowledge, this represents the first systematic analysis of how the postdischarge setting varies from the recommended setting and is hypothesis-generating for developing interventions to optimize poststroke discharge.

Our descriptive findings offer initial evidence of PT and OT discharge recommendations during acute stroke hospitalization, which were mostly static and unchanged during admission. There is an observable trend toward PT and OT recommending an IRF discharge for more patients than the number who discharge to an IRF. A facility discharge requires increased effort to identify a facility, confirm bed availability, and obtain insurance approval, which can prolong the length of hospital admission.^28,29^ This trend could be due, in part, to the timing of the discharge recommendation. It may be challenging to make a recommendation early in an admission versus later, when patients are more medically stable, which could be investigated in future work. In addition, enhancing existing algorithms that identify patients in need of postacute care^30,31^ to indicate the specific type of postacute care (eg, SNF and IRF) may streamline the process and reduce the overrecommending of IRF for those poststroke.

The diverse factors associated with a discrepant discharge poststroke underscore the multilevel complexities that impact patient care and the need to incorporate nonclinical factors in analyses of stroke care.^7,32,33^ That a Sunday discharge was most strongly associated with a discrepant discharge poststroke suggests there could be a “weekend effect” for discharge disposition. Weekend teams may be less familiar with the discharge plan for a patient or less comfortable advocating for the recommended level of care. Similarly, although Medicare Advantage insurance plans require prior authorization for postacute care, this is not required for Medicare fee-for-service. Both Medicare plans were associated with increased odds of a discrepant, lower intensity discharge, suggesting that there may be more factors influencing this than prior authorization alone. There may be an unmeasured bias toward less intensive care settings for Medicare patients who are older, with increased comorbidity, compared with those with commercial insurance. In contrast, a physiatry consultation was associated with significantly lower odds of a discrepant discharge, underscoring their critical role in the discharge process. A physiatrist considers a patient’s current and projected functional status, disease sequelae, and medication regimens, which enables a comprehensive recommendation for the most clinically appropriate discharge setting. Future work could examine the drivers of this discrepancy for Medicare patients and the protective impact of a physiatry consultation on discharge disposition.

Moving forward, interventions that indicate which patients are at the highest risk of a discrepant discharge may facilitate increased advocacy, especially when a more intensive postacute care setting is clinically indicated and support interdisciplinary team communication to reduce nonclinical factors prolonging the admission. Our results can aid in the development of algorithms to identify those most at risk for a discrepant discharge that may need additional care coordination. The use of clinical dashboards to display admissions at higher risk for a discrepant discharge and report discharge metrics can enhance clinical efficiency by offering a centralized location that integrates multiple data sources to improve team communication and care quality, which may be particularly helpful for weekend teams.^34,35^

### Study limitations

There are limitations to consider when interpreting these findings. We did not evaluate the relationship between a discrepant discharge and patient outcomes. As a result, we are unable to make conclusions about whether a discrepant discharge is associated with worse patient outcomes. By including only PT and OT recommendations, we may have missed patients with a speech therapy recommendation that aligned with either PT or OT. There are several patient factors that are known to influence discharge planning poststroke but are not reliably obtained from EHR data. These include existing social support, caregivers, and patient/family preferences for discharge location, all of which were not available for data extraction in this analysis. Making these variables available through the EHR as smart data elements would enable a more robust evaluation of the critical role of social support and patient preference in discharge planning. Similarly, we did not assess speech, cognitive, or swallowing impairments or the Physical Medicine and Rehabilitation discharge recommendation. The Physical Medicine and Rehabilitation recommendation was recorded in narrative text, limiting our ability to reliably obtain this information for this study. We also did not have data regarding bed availability at the next level of care or the number of facility and insurance denials, which may impact the postacute discharge setting. These results may not generalize to subgroups whose PT and OT discharge recommendations differed, which is likely a cohort with a more ambiguous discharge plan. Lastly, data for this study were from an urban academic health system, limiting the generalizability of these findings to other patient populations, especially those from more rural areas who may have less access to some types of postacute care or limited specialized services such as physiatry.

## Conclusions

This study offers initial evidence illustrating the complexity of the discharge process during an acute stroke hospitalization. Although prior work established clinically distinct profiles for patients who discharge to a postacute facility, our findings demonstrate that discrepant discharges are less influenced by clinical presentation and more strongly associated with external, multilevel factors such as day of the week, insurance type, and prestroke mobility. These results underscore the importance of including nonclinical factors in evaluations of poststroke care and identify multiple levers for future interventions to enhance the hospital discharge process poststroke.

## Supplementary Material

Supplemental File

## Figures and Tables

**Fig 1:**
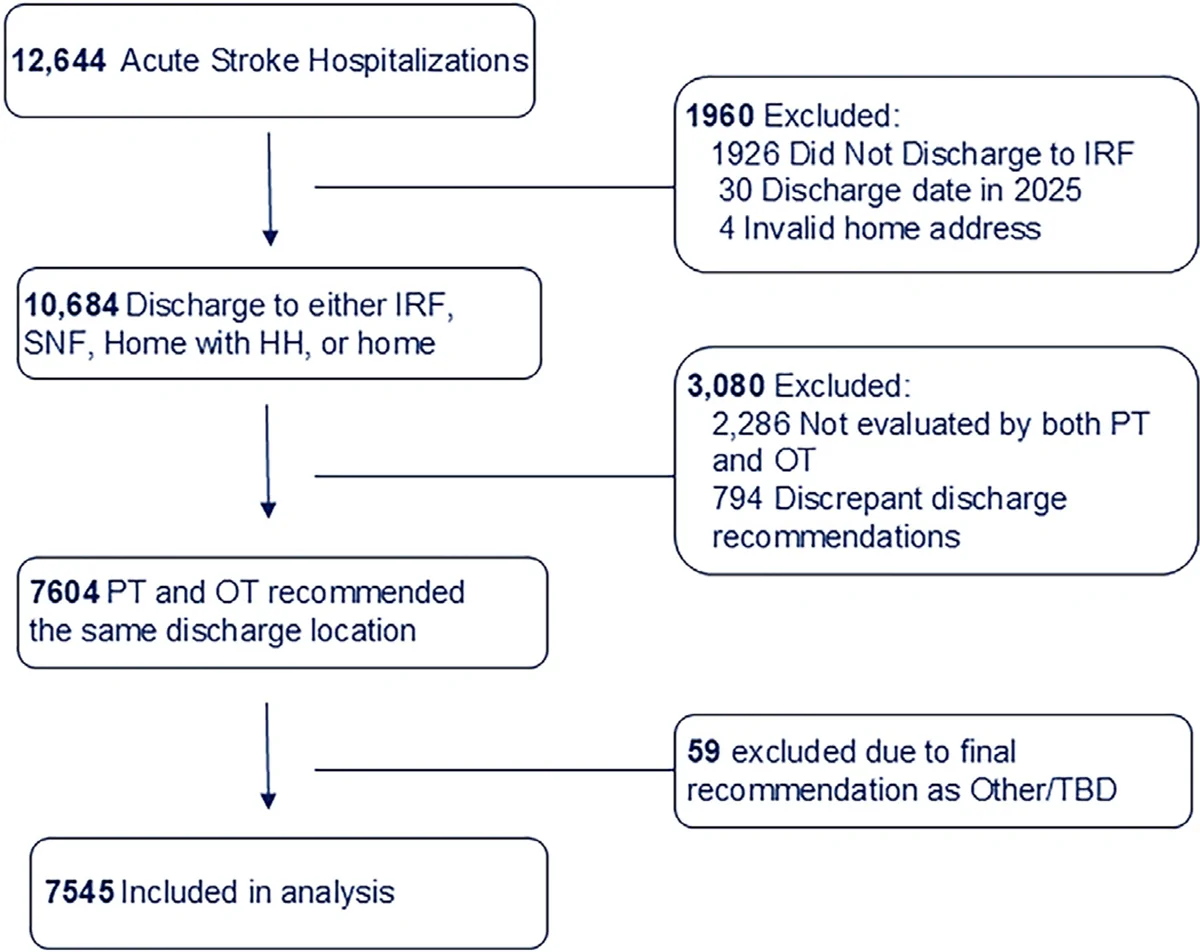
Cohort construction. Abbreviation: TBD, to be determined.

**Fig 2:**
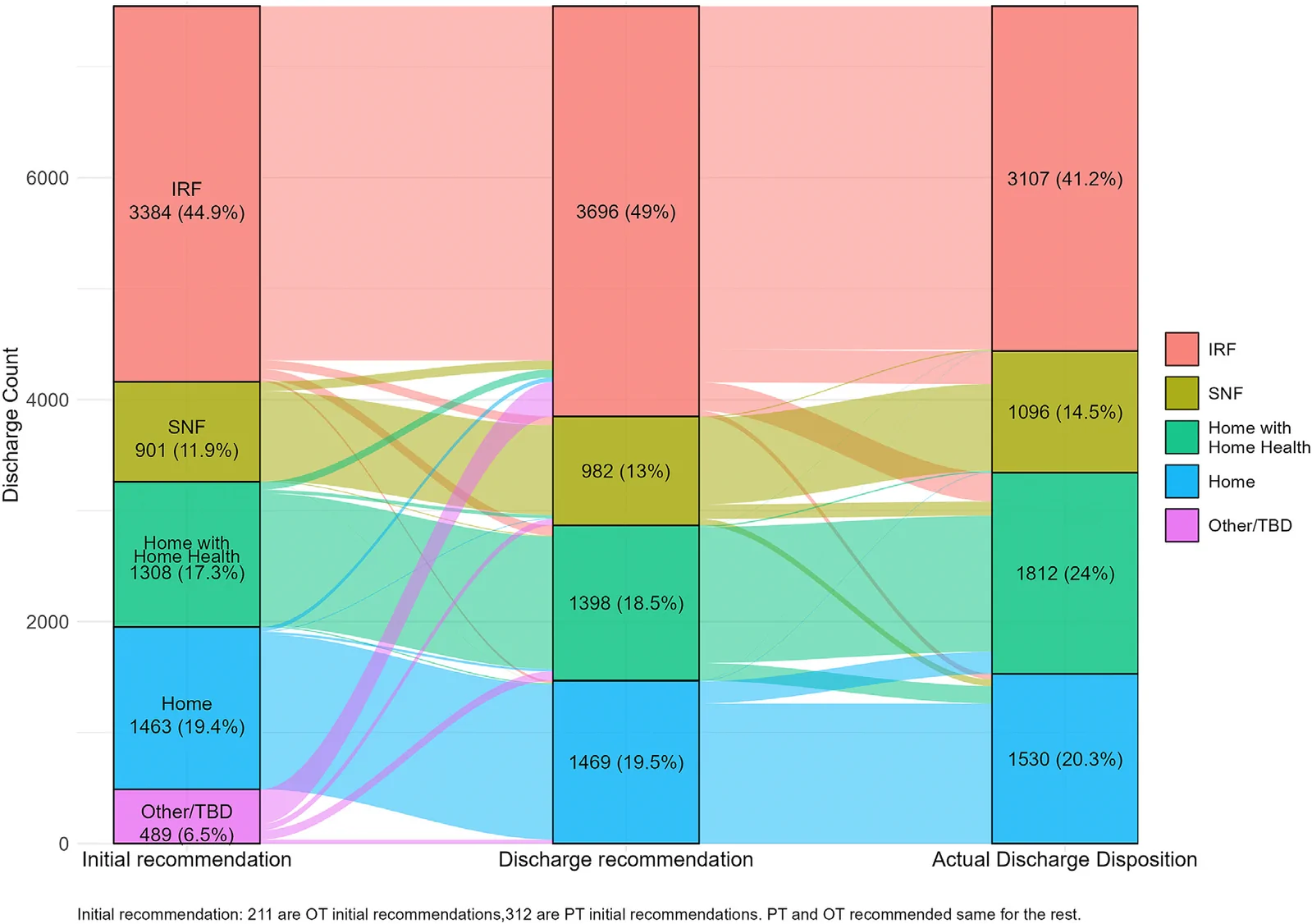
Initial, final, and actual discharge settings. The initial (left) and final (center) PT and OT discharge recommendations and actual discharge disposition (right) by setting. The colored bars represent the proportion of the sample recommended for each setting across the 3 timepoints. If PT and OT did not agree on their initial discharge recommendation, then the PT recommendation at evaluation was used as the initial recommendation. If PT indicated the discharge disposition was to be determined (TBD) or other, then the OT recommendation was used for initial classification.

**Fig 3:**
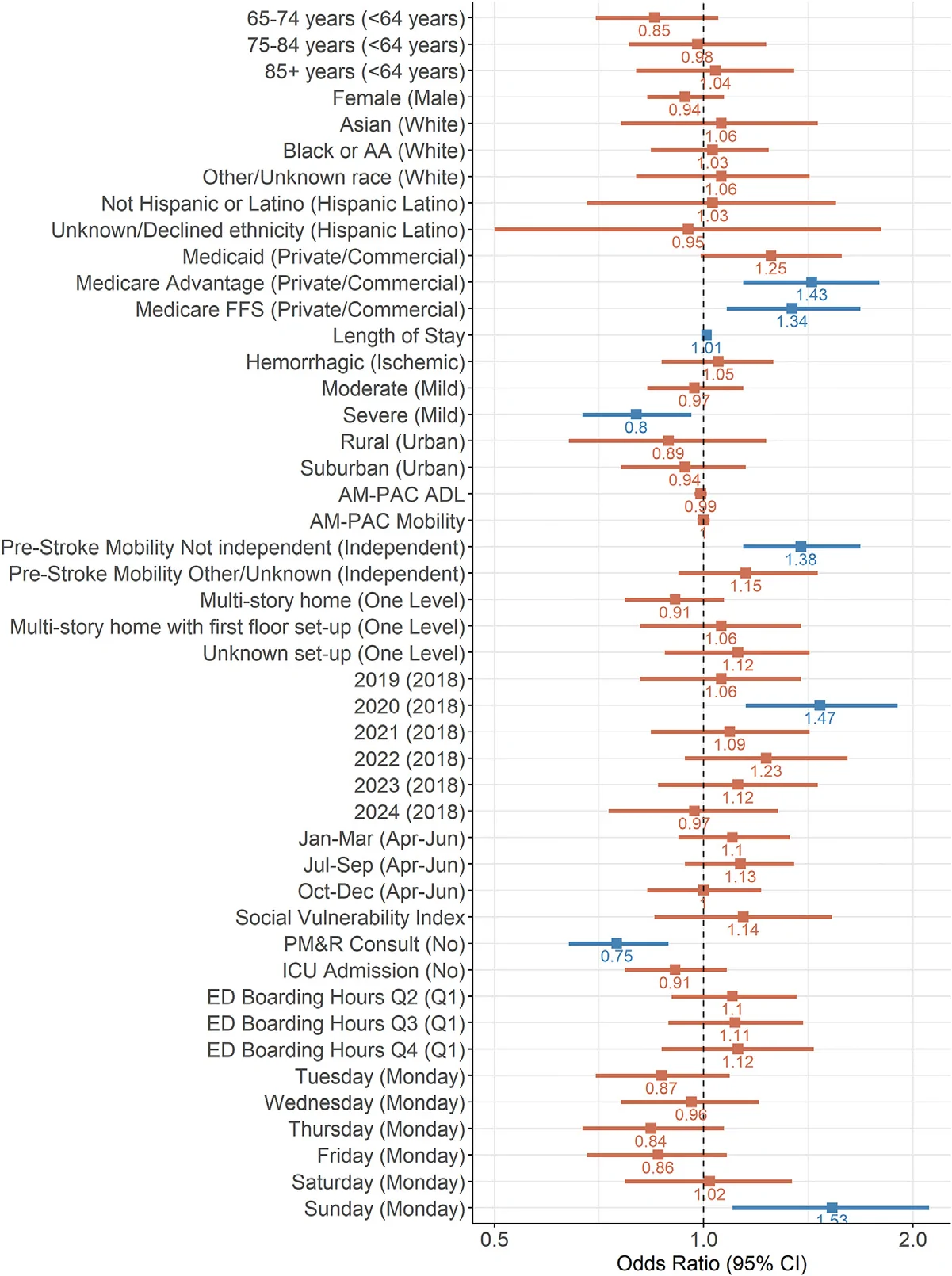
Adjusted odds ratios of sociodemographic, clinical, temporal, and facility variables associated with a discrepant discharge poststroke. The reference group for categorical variables is listed in parentheses, and blue color indicates statistical significance. An odds ratio >1 indicates increased higher odds of a discrepant discharge and <1 indicates lower odds of a discrepant discharge. Abbreviations: FFS, fee-for-service; ICU, intensive care unit; PM&R, Physical Medicine and Rehabilitation.

**Fig 4:**
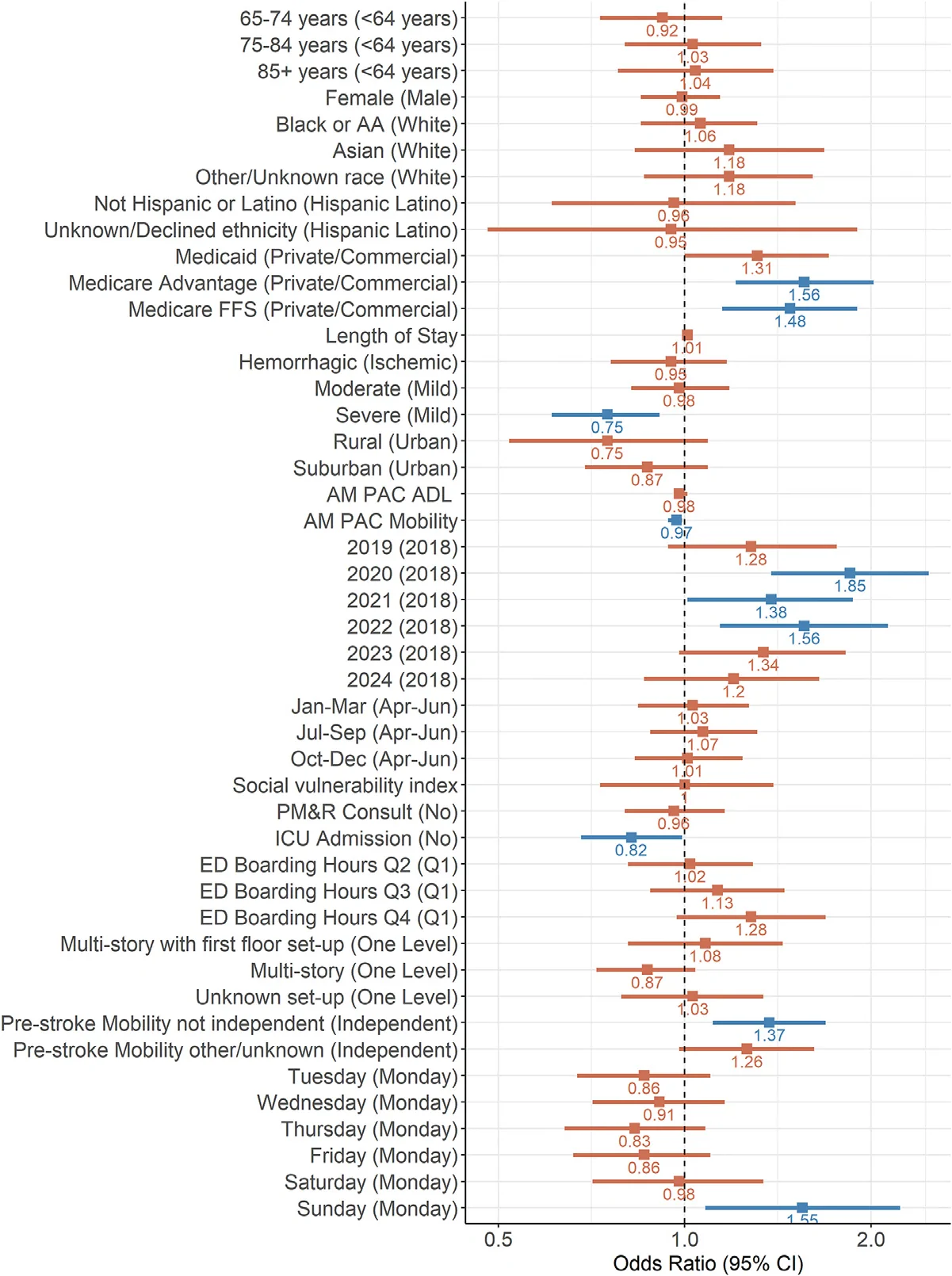
Adjusted odds ratios of sociodemographic, clinical, temporal, and facility variables associated with discharge to a lower intensity setting than what was recommended by PT and OT, compared with those with a matched discharge. The reference group for categorical variables is listed in parentheses, and blue color indicates statistical significance. An odds ratio >1 indicates increased higher odds of discharge to a lower intensity setting, and <1 indicates lower odds of discharge to a lower intensity setting. Abbreviations: FFS, fee-for-service; ICU, intensive care unit; PM&R, Physical Medicine and Rehabilitation.

**Fig 5:**
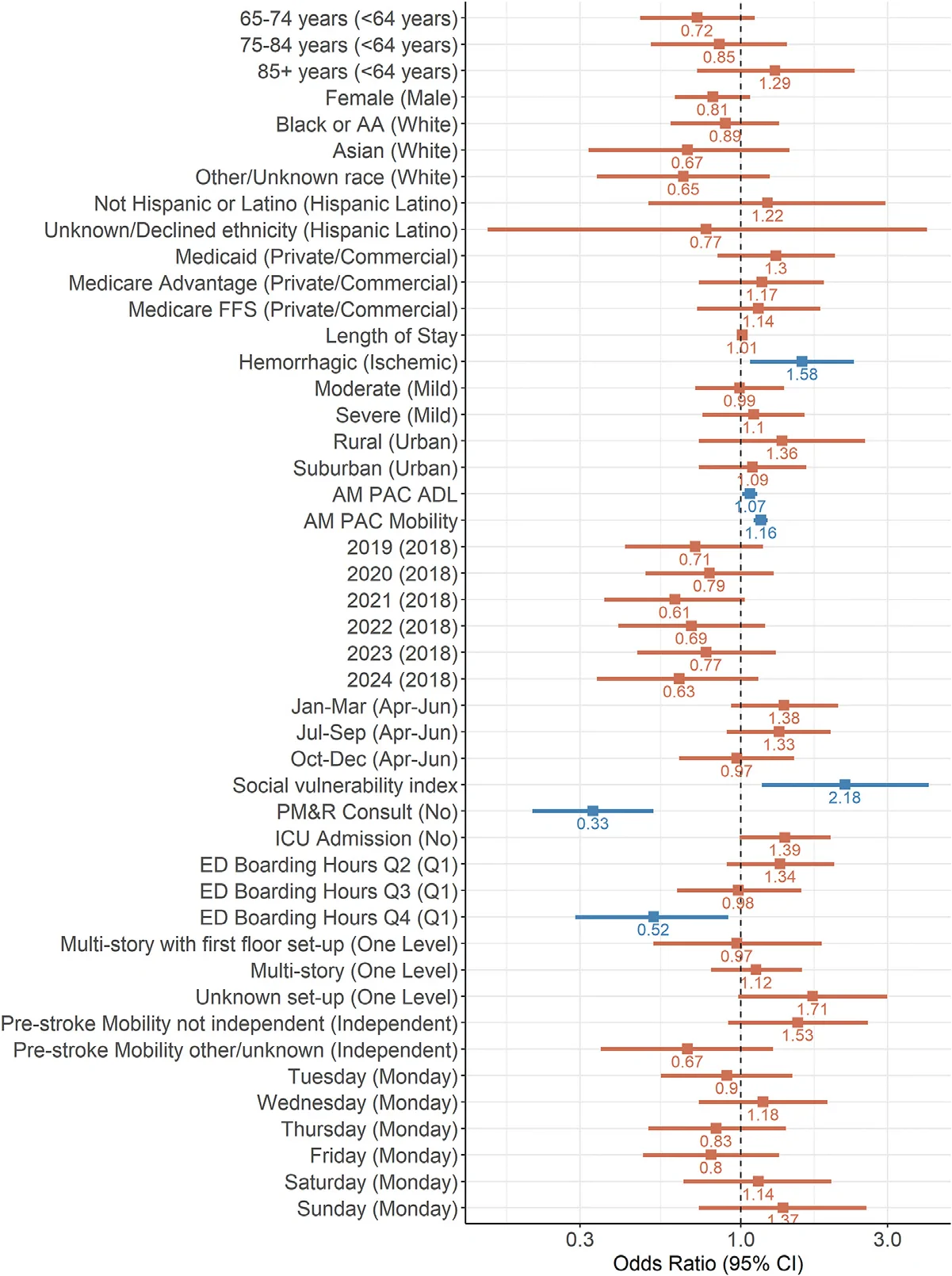
Adjusted odds ratios of sociodemographic, clinical, temporal, and facility variables associated with discharge to a higher intensity setting than what was recommended by PT and OT, compared with those with a matched discharge. The reference group for categorical variables is listed in parentheses, and blue color indicates statistical significance. An odds ratio >1 indicates increased higher odds of a discharge to a higher intensity setting, and <1 indicates lower odds of discharge to a higher intensity setting. Abbreviations: FFS, fee-for-service; ICU, intensive care unit; PM&R, Physical Medicine and Rehabilitation.

**Table 1:** Sample characteristics.

Variable	Overall (N=7545)	Matched Discharge (N=6363)	Discrepant Discharge (N=1182)

Age, mean (SD)	67.9 (14.7)	67.6 (14.7)	69.4 (14.7)
Age, n (%)			
<65 y	2696 (35.7)	2306 (36.2)	390 (33.0)
65–74 y	2197 (29.1)	1879 (29.5)	318 (26.9)
75–84 y	1674 (22.2)	1388 (21.8)	286 (24.2)
85+ y	978 (13.0)	790 (12.4)	188 (15.9)
Sex, n (%)			
Male	3820 (50.6)	3224 (50.7)	596 (50.4)
Female	3725 (49.4)	3139 (49.3)	586 (49.6)
Race, n (%)			
White	3411 (45.2)	2886 (45.4)	525 (44.4)
Black or African American	3169 (42.0)	2651 (41.7)	518 (43.8)
Asian	342 (4.5)	291 (4.6)	51 (4.3)
Other/unknown	623 (8.3)	535 (8.4)	88 (7.4)
Ethnicity, n (%)			
Hispanic Latino	229 (3.0)	194 (3.0)	35 (3.0)
Non-Hispanic or Latino	7173 (95.1)	6043 (95.0)	1130 (95.6)
Unknown/declined	143 (1.9)	126 (2.0)	17 (1.4)
Insurance type, n (%)			
Private/commercial	1893 (25.1)	1668 (26.2)	225 (19.0)
Medicaid	1034 (13.7)	867 (13.6)	167 (14.1)
Medicare FFS	2504 (33.2)	2093 (32.9)	411 (34.8)
Medicare advantage	2114 (28.0)	1735 (27.3)	379 (32.1)
Length of hospital stay	7.90 (10.9)	7.87 (10.2)	8.03 (13.8)
Stroke type, n (%)			
Ischemic	6000 (79.5)	5041 (79.2)	959 (81.1)
Hemorrhagic	1545 (20.5)	1322 (20.8)	223 (18.9)
Stroke Severity Index	5.86 (5.12)	5.94 (5.16)	5.43 (4.87)
Rurality, n (%)			
Urban	3374 (44.7)	2823 (44.4)	551 (46.6)
Suburban	3735 (49.5)	3171 (49.8)	564 (47.7)
Rural	436 (5.8)	369 (5.8)	67 (5.7)
AM-PAC Mobility Score	15.1 (5.96)	15.1 (5.98)	15.2 (5.84)
AM-PACADL Score	17.1 (5.12)	17.1 (5.13)	17.0 (5.07)
Social Vulnerability Index	0.517 (0.311)	0.514 (0.311)	0.532 (0.313)
Received PM&R consult, n (%)	3074 (40.7)	2685 (42.2)	389 (32.9)
ICU admission, n (%)	3000 (39.8)	2604 (40.9)	396 (33.5)

Abbreviations: ADL, activities of daily living; AM-PAC, Activity Measure for Postacute Care; FFS, fee-for-service; ICU, intensive care unit; PM&R, Physical Medicine and Rehabilitation or physiatry.

## Footnotes

a. Supplier R, version 4.3; R Core Team.

## List of abbreviations:

ADL: activities of daily living
AM-PAC: Activity Measure for Postacute Care
aOR: adjusted odds ratio
EHR: electronic health record
HH: home health
IRF: inpatient rehabilitation facility
OT: occupational therapy
PT: physical therapy
SNF: skilled nursing facility
SVI: social vulnerability index
