# Supplementary material for: Factors Associated With Differences in the Recommended Versus Actual Discharge Location After Stroke
Source: Arch Phys Med Rehabil. Author manuscript; Available in PMC 2026 Nov 10. (PMC13750458; doi:10.1016/j.apmr.2026.04.005)
Supplement: Supplementary file 1 [file NIHMS2215404-supplement-Supplemental_File.docx]

**Supplemental File.**

**Table S1**. Stroke ICD-10 codes for cohort construction

**ICD-10 code**

I63.x

I66.x

I67.89x

197.81x

197.82x

G46.x

I60.x

I61.x

I62.x

**Supplemental Figure 1. Unadjusted Trends in discharge disposition by calendar year.** There were changes in discharge patterns in 2020, likely due to the onset of the COVID-19 pandemic, with the most noticeable change in home health use, which persisted over time. Results from a Cochrane Armitage test for trend demonstrated a statistically significant change in home health use and discharges to inpatient rehabilitation by calendar year.

**Table S2.** Odds Ratios and 95% confidence intervals of factors associated with a discrepant discharge after stroke hospitalization

| **Variable** | **Level (Reference)** | **Odd Ratio**  **(95% CI)** | ***P*** |
| --- | --- | --- | --- |
| **Age group** | 65-74 years (<65 years) | 0.85 (0.70, 1.05) | 0.125 |
|  | 75-84 years (<65 years) | 0.98 (0.78, 1.23) | 0.842 |
|  | 85+ years (<65 years) | 1.04 (0.80, 1.35) | 0.765 |
| **Gender** | Female (Male) | 0.94 (0.83, 1.07) | 0.361 |
| **Race** | Asian (White) | 1.06 (0.76, 1.46) | 0.733 |
|  | Black or AA (White) | 1.03 (0.84, 1.24) | 0.803 |
|  | Other/Unknown (White) | 1.06 (0.80, 1.42) | 0.668 |
| **Ethnicity** | Not Hispanic or Latino (Hispanic Latino) | 1.03 (0.68, 1.55) | 0.902 |
|  | Unknown/Declined (Hispanic Latino) | 0.95 (0.50, 1.80) | 0.880 |
| **Insurance type** | Medicaid (Private/Commercial) | 1.25 (0.99, 1.58) | 0.059 . |
|  | Medicare Advantage (Private/Commercial) | 1.43 (1.14, 1.79) | 0.002 ** |
|  | Medicare FFS (Private/Commercial) | 1.34 (1.08, 1.68) | 0.009 ** |
| **Length of hospital stay** | +1 | 1.01 (1.00, 1.01) | 0.028 * |
| **Stroke type** | Hemorrhagic (Ischemic) | 1.05 (0.87, 1.26) | 0.635 |
| **Stroke Severity (SASI)** | Moderate (Mild) | 0.97 (0.83, 1.14) | 0.750 |
|  | Severe (Mild) | 0.80 (0.67, 0.96) | 0.018 * |
| **Rurality** | Rural (Urban) | 0.89 (0.64, 1.23) | 0.473 |
|  | Suburban (Urban) | 0.94 (0.76, 1.15) | 0.550 |
| **AM-PAC ADL score** | +1 | 0.99 (0.97, 1.01) | 0.490 |
| **AM-PAC mobility score** | +1 | 1.00 (0.98, 1.02) | 0.899 |
| **Discharge Year** | 2019 (2018) | 1.06 (0.81, 1.38) | 0.688 |
|  | 2020 (2018) | 1.47 (1.15, 1.90) | 0.002 ** |
|  | 2021 (2018) | 1.09 (0.84, 1.42) | 0.514 |
|  | 2022 (2018) | 1.23 (0.94, 1.61) | 0.135 |
|  | 2023 (2018) | 1.12 (0.86, 1.46) | 0.413 |
|  | 2024 (2018) | 0.97 (0.73, 1.28) | 0.811 |
| **Season** | Jan-Mar (Apr-Jun) | 1.10 (0.92, 1.33) | 0.302 |
|  | Jul-Sep (Apr-Jun) | 1.13 (0.94, 1.35) | 0.195 |
|  | Oct-Dec (Apr-Jun) | 1.00 (0.83, 1.21) | 0.989 |
| **Social vulnerability index percentile** | +1 | 1.14 (0.85, 1.53) | 0.376 |
| **PM&R consult during hospitalization** | Yes (No) | 0.75 (0.64, 0.89) | 0.001 ** |
| **ICU admission during hospitalization** | Yes (No) | 0.91 (0.77, 1.08) | 0.286 |
| **Average ED boarding hours quartile** | 2 (1) | 1.10 (0.90, 1.36) | 0.349 |
|  | 3 (1) | 1.11 (0.89, 1.39) | 0.359 |
|  | 4 (1) | 1.12 (0.87, 1.44) | 0.385 |
| **Prior day average ED boarding hours quartile** | 2 (1) | 0.97 (0.79, 1.19) | 0.780 |
|  | 3 (1) | 1.00 (0.80, 1.26) | 0.980 |
|  | 4 (1) | 0.84 (0.65, 1.08) | 0.164 |
| **Home layout** | Multi-story (One Level) | 0.91 (0.77, 1.07) | 0.237 |
|  | Multi-story with first floor set-up (One Level) | 1.06 (0.81, 1.38) | 0.691 |
|  | Unknown (One Level) | 1.12 (0.88, 1.42) | 0.367 |
| **Pre-admission mobility level** | Not independent (Independent) | 1.38 (1.14, 1.68) | 0.001 ** |
|  | Other/Unknown (Independent) | 1.15 (0.92, 1.46) | 0.225 |
| **Discharge day of week** | Friday (Monday) | 0.86 (0.68, 1.08) | 0.186 |
|  | Saturday (Monday) | 1.02 (0.77, 1.34) | 0.908 |
|  | Sunday (Monday) | 1.53 (1.10, 2.11) | 0.011 * |
|  | Thursday (Monday) | 0.84 (0.67, 1.07) | 0.155 |
|  | Tuesday (Monday) | 0.87 (0.70, 1.09) | 0.233 |
|  | Wednesday (Monday) | 0.96 (0.76, 1.20) | 0.701 |

Data shown as odds ratios and 95% confidence intervals for all variables examined in the primary analysis that evaluated discrepant discharges. Referent group listed in parenthesis.

**Table S3.** Sensitivity Analysis reporting odds ratios and 95% confidence intervals of factors associated with a discrepant discharge after stroke hospitalization

| **Variable** | **Level (Reference)** | **Odd Ratio**  **(95% CI)** | ***P*** |
| --- | --- | --- | --- |
| **Age group** | 65-74 years (<65 years) | 0.89 (0.72, 1.10) | 0.294 |
|  | 75-84 years (<65 years) | 1.01 (0.79, 1.27) | 0.962 |
|  | 85+ years (<65 years) | 1.10 (0.84, 1.45) | 0.469 |
| **Gender** | Female (Male) | 0.97 (0.84, 1.10) | 0.611 |
| **Race** | Asian (White) | 0.97 (0.69, 1.37) | 0.869 |
|  | Black or AA (White) | 1.01 (0.82, 1.23) | 0.941 |
|  | Other/Unknown (White) | 1.05 (0.78, 1.41) | 0.765 |
| **Ethnicity** | Not Hispanic or Latino (Hispanic Latino) | 1.14 (0.73, 1.77) | 0.564 |
|  | Unknown/Declined (Hispanic Latino) | 1.10 (0.56, 2.15) | 0.784 |
| **Insurance type** | Medicaid (Private/Commercial) | 1.24 (0.97, 1.58) | 0.081 . |
|  | Medicare Advantage (Private/Commercial) | 1.38 (1.09, 1.74) | 0.007 ** |
|  | Medicare FFS (Private/Commercial) | 1.28 (1.02, 1.62) | 0.034 * |
| **Length of stay** | +1 | 1.01 (1.00, 1.01) | 0.025 * |
| **Stroke type** | Hemorrhagic (Ischemic) | 1.03 (0.85, 1.26) | 0.740 |
| **SSI category** | Moderate (Mild) | 0.96 (0.81, 1.13) | 0.634 |
|  | Severe (Mild) | 0.77 (0.64, 0.93) | 0.006 ** |
| **Rurality** | Rural (Urban) | 0.89 (0.63, 1.25) | 0.493 |
|  | Suburban (Urban) | 0.97 (0.79, 1.21) | 0.813 |
| **AM-PAC ADL score** | +1 | NA (NA, NA) | NA |
| **AM-PAC mobility score** | +1 | NA (NA, NA) | NA |
| **Year** | 2019 (2018) | 1.08 (0.82, 1.42) | 0.604 |
|  | 2020 (2018) | 1.47 (1.13, 1.90) | 0.004 ** |
|  | 2021 (2018) | 1.12 (0.85, 1.47) | 0.426 |
|  | 2022 (2018) | 1.22 (0.92, 1.61) | 0.173 |
|  | 2023 (2018) | 1.10 (0.83, 1.45) | 0.501 |
|  | 2024 (2018) | 0.95 (0.71, 1.28) | 0.744 |
| **Season** | Jan-Mar (Apr-Jun) | 1.03 (0.84, 1.25) | 0.798 |
|  | Jul-Sep (Apr-Jun) | 1.09 (0.90, 1.31) | 0.367 |
|  | Oct-Dec (Apr-Jun) | 0.96 (0.79, 1.16) | 0.680 |
| **Social vulnerability index percentile** | +1 | 1.20 (0.88, 1.62) | 0.250 |
| **PM&R consult** | Yes (No) | 0.76 (0.64, 0.90) | 0.002 ** |
| **ICU admission** | Yes (No) | 0.92 (0.77, 1.09) | 0.324 |
| **Average ED boarding hours quartile** | 2 (1) | 1.04 (0.84, 1.29) | 0.709 |
|  | 3 (1) | 1.11 (0.88, 1.40) | 0.379 |
|  | 4 (1) | 1.15 (0.88, 1.49) | 0.300 |
| **Prior day average ED boarding hours quartile** | 2 (1) | 0.96 (0.77, 1.19) | 0.684 |
|  | 3 (1) | 0.98 (0.78, 1.24) | 0.880 |
|  | 4 (1) | 0.81 (0.62, 1.05) | 0.110 |
| **Home layout** | Multi-story (One Level) | 0.88 (0.75, 1.04) | 0.146 |
|  | Multi-story with first floor set-up (One Level) | 1.07 (0.81, 1.41) | 0.650 |
|  | Unknown (One Level) | 1.11 (0.86, 1.42) | 0.425 |
| **Pre-admission mobility level** | Not independent (Independent) | 1.37 (1.12, 1.69) | 0.002 ** |
|  | Other/Unknown (Independent) | 1.13 (0.88, 1.44) | 0.335 |
| **Discharge day of week** | Friday (Monday) | 0.89 (0.70, 1.13) | 0.334 |
|  | Saturday (Monday) | 1.12 (0.84, 1.49) | 0.457 |
|  | Sunday (Monday) | 1.58 (1.12, 2.21) | 0.009 ** |
|  | Thursday (Monday) | 0.90 (0.71, 1.15) | 0.407 |
|  | Tuesday (Monday) | 0.90 (0.71, 1.14) | 0.374 |
|  | Wednesday (Monday) | 1.00 (0.79, 1.26) | 0.968 |

Results from the sensitivity analysis that allowed for missing demographic and clinical data.

**Table S4.** Results from the multinomial model evaluating lower or higher intensity discharges, relative to a matched discharge

|  | **Variable** | **Level (Reference)** | **OR**  **(95% CI)** | ***P*** |
| --- | --- | --- | --- | --- |
| **Lower intensity:Matched** | **Age group** | 65-74 years (<65 yrs) | 0.92  (0.73, 1.15) | *0.457* |
| **Higher intensity:Matched** |  | 65-74 years (<65 yrs) | 0.72  (0.47, 1.11) | *0.135* |
| **Lower intensity:Matched** |  | 75-84 years (<65 yrs) | 1.03  (0.80, 1.33) | *0.798* |
| **Higher intensity:Matched** |  | 75-84 years (<65 yrs) | 0.85  (0.51, 1.41) | *0.521* |
| **Lower intensity:Matched** |  | 85+ years (<65 yrs) | 1.04  (0.78, 1.39) | *0.780* |
| **Higher intensity:Matched** |  | 85+ years (<65 yrs) | 1.29  (0.72, 2.34) | *0.394* |
| **Lower intensity:Matched** | **Gender** | Female (Male) | 0.99  (0.85, 1.14) | *0.840* |
| **Higher intensity:Matched** |  | Female (Male) | 0.81  (0.61, 1.07) | *0.139* |
| **Lower intensity:Matched** | **Race** | Black or AA (White) | 1.06  (0.85, 1.31) | *0.619* |
| **Higher intensity:Matched** |  | Black or AA (White) | 0.89  (0.59, 1.33) | *0.566* |
| **Lower intensity:Matched** |  | Asian (White) | 1.18  (0.83, 1.68) | *0.363* |
| **Higher intensity:Matched** |  | Asian (White) | 0.67  (0.32, 1.44) | *0.306* |
| **Lower intensity:Matched** |  | Other/Unknown (White) | 1.18  (0.86, 1.61) | *0.316* |
| **Higher intensity:Matched** |  | Other/Unknown (White) | 0.65  (0.34, 1.24) | *0.190* |
| **Lower intensity:Matched** | **Ethnicity** | Not Hispanic or Latino (Hispanic Latino) | 0.96  (0.61, 1.51) | *0.851* |
| **Higher intensity:Matched** |  | Not Hispanic or Latino (Hispanic Latino) | 1.22  (0.50, 2.95) | *0.662* |
| **Lower intensity:Matched** |  | Unknown/Declined (Hispanic Latino) | 0.95  (0.48, 1.90) | *0.894* |
| **Higher intensity:Matched** |  | Unknown/Declined (Hispanic Latino) | 0.77  (0.15, 4.03) | *0.761* |
| **Lower intensity:Matched** | **Insurance** | Medicaid (Private/Commercial) | 1.31  (1.00, 1.71) | *0.052* |
| **Higher intensity:Matched** |  | Medicaid (Private/Commercial) | 1.30  (0.84, 2.02) | *0.242* |
| **Lower intensity:Matched** |  | Medicare FFS (Private/Commercial) | 1.48  (1.15, 1.90) | ***0.002*** |
| **Higher intensity:Matched** |  | Medicare FFS (Private/Commercial) | 1.14  (0.72, 1.81) | *0.584* |
| **Lower intensity:Matched** |  | Medicare Advantage (Private/Commercial) | 1.56  (1.21, 2.02) | ***<0.001*** |
| **Higher intensity:Matched** |  | Medicare Advantage (Private/Commercial) | 1.17  (0.73, 1.86) | *0.510* |
| **Lower intensity:Matched** | **Length of stay** | +1 | 1.01  (1.00, 1.01) | *0.064* |
| **Higher intensity:Matched** |  | +1 | 1.01  (0.99, 1.02) | *0.202* |
| **Lower intensity:Matched** | **Stroke type** | Hemorrhagic (Ischemic) | 0.95  (0.76, 1.17) | *0.611* |
| **Higher intensity:Matched** |  | Hemorrhagic (Ischemic) | 1.58  (1.07, 2.33) | ***0.020*** |
| **Lower intensity:Matched** | **SSI category** | Moderate (Mild) | 0.98  (0.82, 1.18) | *0.850* |
| **Higher intensity:Matched** |  | Moderate (Mild) | 0.99  (0.71, 1.38) | *0.963* |
| **Lower intensity:Matched** |  | Severe (Mild) | 0.75  (0.61, 0.91) | ***0.005*** |
| **Higher intensity:Matched** |  | Severe (Mild) | 1.10  (0.75, 1.61) | *0.611* |
| **Lower intensity:Matched** | **Rurality** | Suburban (Urban) | 0.87  (0.69, 1.09) | *0.231* |
| **Higher intensity:Matched** |  | Suburban (Urban) | 1.09  (0.73, 1.63) | *0.668* |
| **Lower intensity:Matched** |  | Rural (Urban) | 0.75  (0.52, 1.09) | *0.130* |
| **Higher intensity:Matched** |  | Rural (Urban) | 1.36  (0.73, 2.53) | *0.327* |
| **Lower intensity:Matched** | **AM-PAC ADL score** | +1 | 0.98  (0.96, 1.01) | *0.218* |
| **Higher intensity:Matched** |  | +1 | 1.07  (1.01, 1.13) | ***0.029*** |
| **Lower intensity:Matched** | **AM-PAC mobility score** | +1 | 0.97  (0.94, 0.99) | ***0.002*** |
| **Higher intensity:Matched** |  | +1 | 1.16  (1.10, 1.22) | ***<0.001*** |
| **Lower intensity:Matched** | **Year** | 2019 (2018) | 1.28  (0.94, 1.76) | *0.117* |
| **Higher intensity:Matched** |  | 2019 (2018) | 0.71  (0.42, 1.18) | *0.185* |
| **Lower intensity:Matched** |  | 2020 (2018) | 1.85  (1.38, 2.48) | ***<0.001*** |
| **Higher intensity:Matched** |  | 2020 (2018) | 0.79  (0.49, 1.28) | *0.344* |
| **Lower intensity:Matched** |  | 2021 (2018) | 1.38  (1.01, 1.87) | ***0.041*** |
| **Higher intensity:Matched** |  | 2021 (2018) | 0.61  (0.36, 1.03) | *0.066* |
| **Lower intensity:Matched** |  | 2022 (2018) | 1.56  (1.14, 2.13) | ***0.005*** |
| **Higher intensity:Matched** |  | 2022 (2018) | 0.69  (0.40, 1.20) | *0.188* |
| **Lower intensity:Matched** |  | 2023 (2018) | 1.34  (0.98, 1.82) | *0.066* |
| **Higher intensity:Matched** |  | 2023 (2018) | 0.77  (0.46, 1.30) | *0.331* |
| **Lower intensity:Matched** |  | 2024 (2018) | 1.20  (0.86, 1.65) | *0.282* |
| **Higher intensity:Matched** |  | 2024 (2018) | 0.63  (0.34, 1.14) | *0.127* |
| **Lower intensity:Matched** | **Season** | Jan-Mar (Apr-Jun) | 1.03  (0.84, 1.27) | *0.776* |
| **Higher intensity:Matched** |  | Jan-Mar (Apr-Jun) | 1.38  (0.93, 2.07) | *0.114* |
| **Lower intensity:Matched** |  | Jul-Sep (Apr-Jun) | 1.07  (0.88, 1.31) | *0.478* |
| **Higher intensity:Matched** |  | Jul-Sep (Apr-Jun) | 1.33  (0.90, 1.96) | *0.159* |
| **Lower intensity:Matched** |  | Oct-Dec (Apr-Jun) | 1.01  (0.83, 1.24) | *0.911* |
| **Higher intensity:Matched** |  | Oct-Dec (Apr-Jun) | 0.97  (0.63, 1.49) | *0.900* |
| **Lower intensity:Matched** | **Social vulnerability index percentile** | +1 | 1.00  (0.73, 1.39) | *0.978* |
| **Higher intensity:Matched** |  | +1 | 2.18  (1.17, 4.08) | ***0.015*** |
| **Lower intensity:Matched** | **PM&R consult** | Yes (No) | 0.96  (0.80, 1.16) | *0.701* |
| **Higher intensity:Matched** |  | Yes (No) | 0.33  (0.21, 0.52) | ***<0.001*** |
| **Lower intensity:Matched** | **ICU admission** | Yes (No) | 0.82  (0.68, 0.99) | ***0.043*** |
| **Higher intensity:Matched** |  | Yes (No) | 1.39  (0.99, 1.96) | *0.055* |
| **Lower intensity:Matched** | **Average ED boarding hours quartile** | 2 (1) | 1.02  (0.81, 1.29) | *0.852* |
| **Higher intensity:Matched** |  | 2 (1) | 1.34  (0.90, 2.01) | *0.154* |
| **Lower intensity:Matched** |  | 3 (1) | 1.13  (0.88, 1.45) | *0.338* |
| **Higher intensity:Matched** |  | 3 (1) | 0.98  (0.62, 1.57) | *0.947* |
| **Lower intensity:Matched** |  | 4 (1) | 1.28  (0.97, 1.69) | *0.080* |
| **Higher intensity:Matched** |  | 4 (1) | 0.52  (0.29, 0.91) | ***0.024*** |
| **Lower intensity:Matched** | **Prior day average ED boarding hours quartile** | 2 (1) | 0.95  (0.76, 1.20) | *0.682* |
| **Higher intensity:Matched** |  | 2 (1) | 1.08  (0.71, 1.63) | *0.732* |
| **Lower intensity:Matched** |  | 3 (1) | 0.97  (0.76, 1.25) | *0.843* |
| **Higher intensity:Matched** |  | 3 (1) | 1.14  (0.72, 1.83) | *0.577* |
| **Lower intensity:Matched** |  | 4 (1) | 0.78  (0.59, 1.03) | *0.081* |
| **Higher intensity:Matched** |  | 4 (1) | 1.08  (0.63, 1.85) | *0.788* |
| **Lower intensity:Matched** | **Home layout** | Multi-story with first floor set-up (One Level) | 1.08  (0.81, 1.44) | *0.610* |
| **Higher intensity:Matched** |  | Multi-story with first floor set-up (One Level) | 0.97  (0.52, 1.83) | *0.924* |
| **Lower intensity:Matched** |  | Multi-story (One Level) | 0.87  (0.72, 1.04) | *0.114* |
| **Higher intensity:Matched** |  | Multi-story (One Level) | 1.12  (0.80, 1.58) | *0.503* |
| **Lower intensity:Matched** |  | Unknown (One Level) | 1.03  (0.79, 1.34) | *0.822* |
| **Higher intensity:Matched** |  | Unknown (One Level) | 1.71  (0.98, 2.99) | *0.059* |
| **Lower intensity:Matched** | **Pre-admission mobility level** | Not independent (Independent) | 1.37  (1.11, 1.69) | ***0.003*** |
| **Higher intensity:Matched** |  | Not independent (Independent) | 1.53  (0.91, 2.59) | *0.112* |
| **Lower intensity:Matched** |  | Other/Unknown (Independent) | 1.26  (0.98, 1.62) | *0.069* |
| **Higher intensity:Matched** |  | Other/Unknown (Independent) | 0.67  (0.35, 1.27) | *0.223* |
| **Lower intensity:Matched** | **Discharge day of week** | Tuesday (Monday) | 0.86  (0.67, 1.10) | *0.225* |
| **Higher intensity:Matched** |  | Tuesday (Monday) | 0.90  (0.55, 1.47) | *0.671* |
| **Lower intensity:Matched** |  | Wednesday (Monday) | 0.91  (0.71, 1.16) | *0.437* |
| **Higher intensity:Matched** |  | Wednesday (Monday) | 1.18  (0.73, 1.91) | *0.489* |
| **Lower intensity:Matched** |  | Thursday (Monday) | 0.83  (0.64, 1.08) | *0.167* |
| **Higher intensity:Matched** |  | Thursday (Monday) | 0.83  (0.50, 1.40) | *0.491* |
| **Lower intensity:Matched** |  | Friday (Monday) | 0.86  (0.66, 1.10) | *0.229* |
| **Higher intensity:Matched** |  | Friday (Monday) | 0.80  (0.48, 1.33) | *0.386* |
| **Lower intensity:Matched** |  | Saturday (Monday) | 0.98  (0.71, 1.34) | *0.881* |
| **Higher intensity:Matched** |  | Saturday (Monday) | 1.14  (0.65, 1.97) | *0.652* |
| **Lower intensity:Matched** |  | Sunday (Monday) | 1.55  (1.08, 2.23) | ***0.019*** |
| **Higher intensity:Matched** |  | Sunday (Monday) | 1.37  (0.73, 2.56) | *0.331* |

Results presented as odds ratios (95% CI). The reference group were patients with a matched discharge.
